# Dietary microalgal-fabricated selenium nanoparticles improve Nile tilapia biochemical indices, immune-related gene expression, and intestinal immunity

**DOI:** 10.1186/s12917-024-03966-4

**Published:** 2024-03-18

**Authors:** Eman Zahran, Samia Elbahnaswy, Fatma Ahmed, Engy Risha, Abdallah Tageldein Mansour, Arwa sultan Alqahtani, Walaa Awadin, Mahmoud G. El Sebaei

**Affiliations:** 1 Department of Aquatic Animal Medicine, Faculty of Veterinary Medicine, Mansoura University, Mansoura, 35516 Egypt; 2 Department of Zoology, Faculty of Science, Sohag University, Sohag, 82524 Egypt; 3 Department of Clinical Pathology, Faculty of Veterinary Medicine, Mansoura University, Mansoura, 35516 Egypt; 4 Animal and Fish Production Department, College of Agricultural and Food Sciences, King Faisal University, P.O. Box 420, Al-Ahsa, 31982 Saudi Arabia; 5 Fish and Animal Production Department, Faculty of Agriculture (Saba Basha), Alexandria University, Alexandria, 21531 Egypt; 6 Department of Chemistry, College of Science, Imam Mohammad Ibn Saud Islamic University (IMSIU), P.O. Box, Riyadh, 9095011623 Saudi Arabia; 7 Department of Pathology, Faculty of Veterinary Medicine, Mansoura University, Mansoura, 35516 Egypt; 8 Department of Biomedical Sciences, College of Veterinary Medicine, King Faisal University, Al-Ahsa, 31982 Saudi Arabia; 9 Department of Biochemistry and Molecular Biology, Faculty of Veterinary Medicine, Mansoura University, Mansoura, 35516 Egypt

**Keywords:** Fish, Nanoparticles, Health indicators, Gut integrity

## Abstract

**Background:**

Feed supplements, including essential trace elements are believed to play an important role in augmenting fish immune response. In this context, selenium nanoparticles (SeNPs) in fish diets via a green biosynthesis strategy have attracted considerable interest. In this investigation, selenium nanoparticles (SeNPs, 79.26 nm) synthesized from the green microalga *Pediastrum boryanum* were incorporated into Nile tilapia diets to explore its beneficial effects on the immune defense and intestinal integrity, in comparison with control basal diets containing inorganic Se source. Nile tilapia (No. 180, 54–57 g) were fed on three formulated diets at concentrations of 0, 0.75, and 1.5 mg/kg of SeNPs for 8 weeks. After the trial completion, tissue bioaccumulation, biochemical indices, antioxidant and pro-inflammatory cytokine-related genes, and intestinal histological examination were analyzed.

**Results:**

Our finding revealed that dietary SeNPs significantly decreased (*P* < 0.05) serum alkaline phosphatase (ALP), lactate dehydrogenase (LDH), and cholesterol, while increasing (*P* < 0.05) high-density lipoproteins (HDL). The Se concentration in the muscle tissues showed a dose-dependent increase. SeNPs at a dose of 1.5 mg/kg significantly upregulated intestinal interleukin 8 (*IL-8*) and interleukin 1 beta *(IL-1β)* gene transcription compared with the control diet. Glutathione reductase (*GSR*) and glutathione synthetase (*GSS*) genes were significantly upregulated in both SeNPs-supplemented groups compared with the control. No apoptotic changes or cell damages were observed as indicated by proliferating cell nuclear antigen (*PCNA*) and *caspase*-3 gene expression and evidenced histopathologically. SeNPs supplementation positively affects mucin-producing goblet cells (GCs), particularly at dose of 1.5 mg/kg.

**Conclusion:**

Therefore, these results suggest that Green synthesized SeNPs supplementation has promising effects on enhancing Nile tilapia immunity and maintaining their intestinal health.

## Introduction

With aquaculture intensification and the increasing probability of disease outbreaks, new strategies have been implemented to overcome these challenges [1]. One of these strategies is to use feed supplementation, and recently nanoparticles (NPs) dietary supplementations have been paid great attention owing to their greater benefits for fish [2]. Green biosynthesis of metals NPs is more promising than the chemically synthesized ones since a biogenic approach requires non-toxic solvents, low temperatures, and inexpensive biodegradable reducing agents. In general, nanoparticles (NPs) produced by biological organisms show better physicochemical characteristics, such as smaller size, large surface area, high stability, and minimal cytotoxicity [3, 4]. In addition, they are not harmful to the environment because living organisms, such as fungi, algae, bacteria, and plants, can reduce and stabilize metals used as a method of detoxification [5, 6].

Selenium (Se) is a dietary trace mineral involved in the metabolism of living organisms. The optimal necessities of Se fluctuated from 0.15 to 0.7 mg/ kg in many fish species [7]. It plays a pivotal role in antioxidant resistance and the regulation of metabolic pathways such as thyroid hormones, cellular growth, and immune capacity [8]. It also acts as a shelter against oxidative stress because it is essential for the production of selenoenzymes such as glutathione peroxidase (GPx) and selenocysteine [9]. The primary consequences of selenium deficiency in fish include increased susceptibility to pathogens, growth scarcity, immunosuppression, and inflammatory diseases [10, 11]. However, high Se concentrations and long-term Se supplementation are associated with toxicity [12]. In the natural environment, selenium is present in inorganic forms, such as selenite Se (IV) and selenate Se (VI) ions, and as an organic species with direct Se-C bonds (methylated compounds, selenoamino acids, and selenoproteins) [13]. This immensity of Se’s benefits rendered its incorporation into fish diets via green biosynthesis as nano-selenium essential [5, 13, 14]. Moreover, as a component of proteins such as selenoproteins, Se can improve the process of digestion, leading to an increase in goblet cells, which is linked to mucosal immunity [15]. Mucosal barriers and the antioxidant system have a pivotal roles of aquatic animals' disease resistance and are classified as components of the innate immune system [16].

SeNPs Biosynthesis using plants had been investigated in many literature, however, using microalgae to green synthesize SeNPs is more preferable, considering their rapid growth and ability to double their mass faster than higher plants, besides; their capabilities to reduce metal ions [17, 18], due to the formation of biomolecular complexes with metal-chelating biomolecules in algal extracts (e.g., polysaccharides, peptides, and pigments) for capping metal nanoparticles [19, 20]. In this context, members of the genus *Pediastrum* (Sphaeropleales, Hydrodictyaceae), unicellular and colonial chlorophytes, are promising microalgae for biotechnological, food, industrial, and pharmaceutical applications [21, 22]. The most widely distributed species in eutrophic freshwater and sediments from the Cretaceous of Egypt is *Pediastrum boryanum* (*P. boryanum,* Turpin) [23]. The green microalga *P. boryanum* produces higher levels of secondary metabolites, such as carotenoids, polyunsaturated fatty acids, vitamins, carotenoid pigments, and polyphenols, demonstrating a notable inhibitory effect on lipid peroxidation [24]. Prior studies have examined the impacts of SeNPs produced from various microalgae strains, including Spirulina platensis-SeNPs and numerous cyanobacterial strains [25, 26]. Nevertheless, no research has been conducted on the SeNPs synthesized using the green microalga *P. boryanum.* Therefore, it is noteworthy to explore how might these NPs-based microalgae contribute to augment Nile tilapia immune response and thus control diseases in tilapia farming system.

The farmed Nile tilapia, *Oreochromis niloticus,* mainly contributes to the animal protein supply and food safety for millions of Egyptian populations, as this fish was produced nationally by 66% of the total cultured fish species and 43% of the total fish consumption in Egypt in 2019 [27, 28]. Therefore, we aimed to explore the potential impact of *P. boryanum-derived* biosynthesized SeNPs on Nile tilapia’s immune response via selected biomarkers, including serum biochemical parameters, and sets of selected functional related-genes expression, besides, intestinal integrity. To the best of our knowledge, *P. boryanum* is a novel green microalga that has not previously been studied in fish.

## Material and methods

### Ethical approval

The experiment was conducted following the protocol involving the use of animals that were approved by the Mansoura University Animal Care and Use Committee (VM.R.23.12.135). All fish handling procedures and regulations followed the ARRIVE guidelines for Animal Care and Use. Furthermore, all relevant organisational and government rules and regulations governing the ethical use of experimental animals were followed. Written informed owner consent has been obtained in this study.

### Preparation of *Pediastrum boryanum* extract

The selected microalga, *P. boryanum* powder (National Research Centre, Cairo, Egypt), was subjected to extraction according to previously described methods [29], with a slight modification to be convenient for the newly invented device, namely the El-Ghamry and El-Khateeb Bio-Nano Apparatus. This instrument consists of ten units, starting from the solvent unit to the extraction, biosynthesis, and control panel units, as detailed in a previous study [30]. Briefly, the microalgal plant (100 g) was dissolved in the first solvent unit containing 1 L distilled water (DW) using a magnetic stirrer (Lss Egypt, Cairo, Egypt). The dissolved solution was boiled in the extraction unit at 70 °C for 2 h. The *P. boryanum* extract was filtered through Whatman filter paper using a Büchner funnel. The volume of the filtrates was adjusted to 1 L in a volumetric bottle with deionized DW. Finally, part of this extract was directly transferred to the biosynthesis unit to further generate Se nanoparticles.

### Synthesis of selenium nanoparticles (SeNPs)

The biological synthesis of the metal nanoparticles was performed according to previously reported procedures [6, 31] with some modifications to fit the newly invented apparatus [30]. This step was performed in the biosynthesis unit, in which an aqueous solution of 1 L of selenium (IV) oxide (Sublimed, Merck, Darmstadt, Germany) was added slowly dropwise to 1 L of the prepared algal extract solution under magnetic stirring. After continuous stirring of the mixture for an extra two hours at room temperature, the mixed solution was automatically transferred to the irradiation unit for UV irradiation using a reduction factor lamp (Vilber Lourmat-6. LC, France) at a wavelength (λ = 254 nm) for 20 min, according to a previously reported method [32]. The synthesized nanoparticles were filtered through Whatman no. 1 filter paper (Whatman International Ltd., Kent, UK) and then transferred to the product storage unit, where the final product of Se nanoparticles was stored at − 18 °C for further experiments.

### Characterization of SeNPs

The morphological features (*e.g*., particle size, shape, and surface nature) of the SeNPs were examined using transmission electron microscopy (TEM) (JEOL TEM-2100, Tokyo, Japan) at the Electron Microscope Unit, Mansoura University, Egypt, as described in a previous study [6]. A drop of the prepared solution was spread onto a carbon-coated copper grid, which was then dried at room temperature and photographed under a microscope at 200 nm magnification value. The samples were subjected to crystallographic analysis using powder X-ray diffraction (XRD). Scanning mode X-ray diffraction patterns were captured using a Bruker D2 Phaser analytical instrument set at 30 kV and 10 mA current with Cu K radiation (λ = 1.54060 Ω). The intensities ranging from 5° to 79.93° were measured at two angles. A comparison was made between the diffraction intensities and the standard JCPDS files. The surface charge and stability of the prepared selenium nanoparticles were characterized using a zeta potential analyzer (Malvern Zetasizer® Version 2.3, Kassel, Germany) in the same Electron Microscope Unit, which depends on electrophoretic light scattering [33].

### Experimental rearing and feeding regimes of Nile tilapia

The basal ingredients of the fish feed were prepared at the laboratory of the Department of Nutrition, Faculty of Veterinary Medicine, Mansoura University. The SeNPs were individually incorporated into the basal diet at three different concentrations, 0, 0.75, and 1.5 mg/kg. The three diets were formulated according to NRC [34], as presented in Table 1. The experimental groups were as follows: the control group was fed a basal diet (containing Se in the mineral mix (inorganic form Na_2_Seo_3_ as 0.2 mg/Kg diet), SeNPs_0.75_ group was fed a mineral premix Se- free diet supplemented with SeNPs at 0.75 mg/kg body weight, and SeNPs_1.5_ group was fed a mineral premix Se- free diet supplemented with SeNPs at 1.5 mg/kg body weight. SeNPs suspensions at concentrations of 0.75 and 1.5 SeNPs mg/kg feed were gradually added and thoroughly mixed with the ingredients of the basal diet. All dietary components were mixed with gelatin, and sterilized water was added until the formation of a stiff paste. The paste was pelleted into 3-mm-diameter pellets using a meat mincer (ME605131 1600-Watt, Moulinex, Groupe SEB, France). Finally, the pellets were oven-dried at 50 °C for 24 h before being placed in a plastic bag and stored at 4 °C until use.

**Table 1:** Formulation and proximate composition of basal and experimental diets

Ingredients (%)	Control	SeNPs_0.75_	SeNPs_1.5_
Yellow corn	19.5	19.5	19.5
Soybean meal	20	20	20
Fish meal	20	20	20
Corn gluten	3	3	3
Gelatin	2	2	2
Sunflower oil	3.50	3.50	3.50
Wheat bran	30.16	30.16	30.16
Minerals and vitamins premix^a^	1	1	1
Salt	0.30	0.30	0.30
Vitamin C	0.12	0.12	0.12
Dicalcium phosphate	0.10	0.10	0.10
Methionine	0.32	0.32	0.32
SeNPs (mg/kg)	0	0.75	1.5
**Proximate analysis (% dry matter basis)**			
Crude Protein*	32.04	32.04	32.04
Lipid*	7.06	7.06	7.06
Ca*	1.17	1.17	1.17
P*	0.53	0.53	0.53
DE (Digestable Energy)** (kcal/kg)	3016	3016	3016
Se content (mg/Kg)	0.2	0.75	1.5

^a^The levels of the micro minerals &vitamins for tilapia are covered by supplementation of trace minerals & vitamins premixes as recommended by NRC (2011). Vitamins premix (IU or mg/kg diet); vit. A 5000, Vit.D3 1000, vit. E 20, vit. k3 2, vit. B1 2, vit. B2 5, vit. B6 1.5, vit. B12 0.02, Pantothenic acid 10, Folic acid 1, Biotin 0.15, Niacin 30. Mineral mixture (mg/kg diet); Fe 40, Mn 80, Cu 4, Zn 50, I 0.5, Co 0.2 & Se 0.2. *Analysed. **DE calculated according to M Jobling [92]The Gross energy calculated according to NRC (2011), as follow: CP × 5.64 + EE × 9.44 + NFE × 4.11; whereas [Nitrogen free extract (NFE) = [100-(CP + EE + CF + Ash)]. The DE was calculated according to Jobling, (1983), as follows: Digestible energy = gross energy X 0.75

A total of 180 apparently healthy Nile tilapia, with an average body weight of 54–57 g, were cultured in a private fish farm, Lake Manzala, Bahr El-Baqar drain, Egypt, and were used in this study. The fish were randomly distributed into three experimental groups in triplicate (20 fish/hapa). They were allocated into nine hapas (200 × 500 × 100 cm^3^, 10 m^3^) for each experiment, where the water quality parameters were monitored biweekly at a temperature of 26 °C, the dissolved oxygen ranged from to 6.7–6.9 mg/liter, and the pH level was adjusted to 7.3 ± 0.2. The water exchange (10%) was performed daily. During the acclimatization period, fish were fed a basal diet twice (at 9 a.m. and 4 p.m., respectively) per day at 3% of their biomass (on a dry matter basis). The fish were weighed every 2 weeks to readjust the feeding quantity. The experiment lasted for 8 weeks.

### Serum and tissue sampling

After the fish were anesthetized with clove oil at 60 mg/L, blood samples were collected from the caudal vessels of six fish per group using non-heparinized disposable syringes for serum separation, centrifuged at 1198 × g for 15 min at 4 °C, and stored at – 20 °C for analysis of biochemical parameters and lipid profile. For digestive enzyme analysis, part of the intestinal tissue was separated, washed many times with cold 0.9% NaCl solution, and stored at – 80 °C. Next, 100 mg of the intestine was collected in Eppendorf tubes containing 500 µL of RNA later® (Sigma) solution and stored at – 20 °C for estimation of gene expression. In addition, intestinal samples were dissected and placed in a 10% neutral buffered formaldehyde solution for histomorphometric analyses.

### Determination of serum biochemical assays

Serum alkaline phosphatase (ALP, Elitech Group Inc., 55,230, Envoy500, California, USA) and lactate dehydrogenase (LDH, Elitech Group Inc., 55,395, USA) activities were quantitatively determined according to the manufacturers’ instructions. In addition, the lipoprotein profile, including cholesterol, triglycerides (TG), low-density lipoproteins (LDL), and high-density lipoproteins (HDL), were measured calorimetrically using diagnostic reagent kits (SPINREACT Diagnostics, S.A./S.A.U Ctra., Santa Coloma, Spain), according to the standard protocol for their specific pamphlets [35–38].

### Estimation of Se mineral contents in fish diets and musculature

The Se content of the test diets and muscles was assessed by the digestion of samples in nitric acid (AOAC 1998). Samples were collected at random and dried for 48 h at 105 °C. The samples were digested with concentrated H_2_SO_4_. Se concentrations in the fish musculature were determined using an atomic absorption spectrophotometer (PG990, UK) following the standard method [39].

### RNA extraction, complementary DNA synthesis, and qRT-PCR

Total RNA was manually extracted from 100 mg of each intestinal sample from each group (control, SeNPs0.75, SeNPs1.5) using a handheld homogenizer to homogenize the tissue immersed in one mL Genzol™ (Geneaid Biotech Ltd., Taiwan) without DNase treatment. The pellet was dissolved in TE buffer (pH 8.0) as described previously [40]. The RNA quantity was estimated using a NanoDrop spectrophotometer (Q5000/Quawell, Massachusetts, USA). Complementary DNA (cDNA) containing 1 μg of total RNA was synthesized using a TOPscript™ RT DryMIX(dT18) cDNA Synthesis Kit (Enzynomics Co Ltd, Daejeon, Republic of Korea) according to the manufacturer's protocol. The specific primers used to amplify the selected genes of Nile tilapia with antioxidant genes: Glutathione peroxidase (*GPx*), Glutathione-S-transferase (*GST*), Glutathione reductase (*GSR*), and Glutathione synthetase (*GSS*); pro-inflammatory genes, Tumor necrosis factor-alpha (*TNF-α*), Interleukin 8 (*IL-8*), and Interleukin 1 beta (*IL-1β*); anti-inflammatory genes, Transforming Growth Factor-β (*TGF-β*); apoptotic and regulatory-related genes, proliferating cell nuclear antigen (*PCNA*) and *caspase*-3, in addition to the *β-actin* as a housekeeping gene were described elsewhere [41–43]. The QuantStudio™ 1 Real-Time PCR System (Applied Biosystems™ Thermo Fisher Scientific, USA) was used to quantify the expression of genes using Solg™ 2X Real-Time PCR Smart mix (Including SYBR® Green) (SolGent Co., Ltd. Yuseong-gu, Daejeon, Korea). The thermocycling conditions were as follows: 95 °C for 20 s, followed by 40 cycles of denaturation at 60 °C for 40 s, and elongation at 72 °C for 30 s.

### Histochemical differentiation of the intestinal mucin-producing goblet cells

The intestinal tissue samples were fixed in 10% neutral buffered formalin for 24 h, embedded in paraffin wax, and sectioned at 5 µm. Selected slides were routinely stained with Hematoxylin and Eosin (H&E), according to KS Suvarna, C Layton and JD Bancroft [44] and were examined under a light microscope (Olympus CX 31). Goblet cells (GCs) in the intestine were semi-quantified as previously described by Ahmed et al. [45], with minor modifications. In brief, intestinal samples were stained with Alcian Blue AB (pH 2.5) and Periodic-Acid Schiff (PAS) double staining for GCs differentiation, according to Padra et al. [46]. Mucin-free and mucin-filled GCs in intact villi along 5000 µm length of the mucosal epithelium were counted in triplicate slides per treated group [47]. The differential count of mucin-producing GCs depended on their visible color under a light microscope (Olympus CX 31). Acid mucin-producing GCs were stained blue with AB (pH 2.5), neutral mucin-producing GCs were stained pink with PAS, mixed mucin-producing GCs were double stained and appeared purple, whereas mucin-free GCs were negatively stained. Triplicate blinded fields (40 ×) per examined section were surveyed, and the obtained data were expressed as mean percentage ± SD.

### Statistical analysis

The data were first checked for normality and homogeneity using Kolmogorov–Smirnov and Levene's tests, respectively. One-way analysis of variance (ANOVA) was used to determine the significance of the group variables using GraphPad® statistics package version 8.4.2. (GraphPad Software, Inc., USA). Individual fold-change values were normalized and anchored to the lowest value recorded in each data set before Log2 transformed, as previously described [48]. The significance level was set at *P* < 0.05 (*), *P* < 0.01 (**), and *P* < 0.001 (***). All data are presented as mean ± SEM.

## Results

### Characterization of SeNPs

**Fig. 1:**
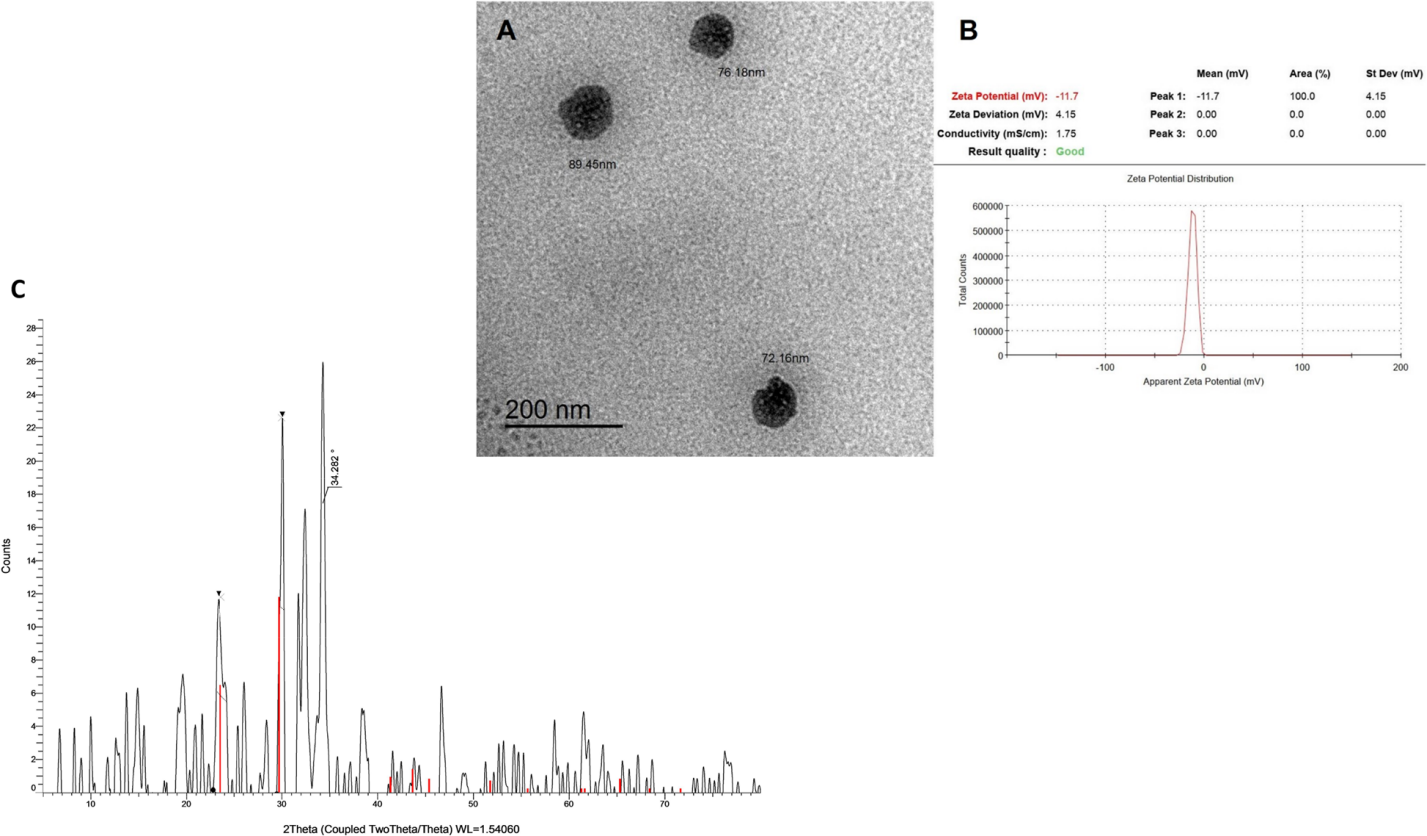
Transmission electron microscope micrographs and zeta potential graphs of the prepared selenium nanoparticles of the *Pediastrum boryanum* extract. **A** TEM micrographs and size distributions for biosynthesized selenium nanoparticles by *P. boryanum* extract at a 200 nm magnification value. **B** Zeta potential of the prepared nano-selenium synthesized by *P. boryanum* extract. **C** X-ray diffraction (XRD) pattern of SeNPs

The morphology and size of the prepared selenium nanoparticles from *P. boryanum* were determined using TEM. The formed nanoparticles exhibited spherical and tetragonal shapes at a higher spatial resolution (200 nm). The size of the selenium particles ranged from 72.16 nm to 89.45 nm (Fig. 1A). The selenium particles synthesized with the algal extract had zeta potentials of − 11.7 mV (Fig. 1 B), showing a higher degree of stability, as nanoparticles had zeta potential values greater than − 25 mV. Additionally, the results were interpreted. The nature of a double layer of ions on the surface of the nanoparticles allows more diffusion into the solution. The XRD pattern of the selenium dioxide nanoparticles (Fig. 1C) shows peaks that correspond to the atomic planes in the crystal structure. The predominant phase of Se dioxide is α-SeO_2_, which has a monoclinic crystal structure. α-SeO_2_ nanoparticles exhibit several peaks, the strongest peaks in the XRD pattern of α-SeO_2_ nanoparticles are at 21°, 29°, and 34 suggesting that the α-SeO_2_ nanoparticles in the sample are orientated parallel to the sample surface, with planes (110), (121), (021), and (201).

### Selenium content in fish diet and musculature

**Fig. 2:**
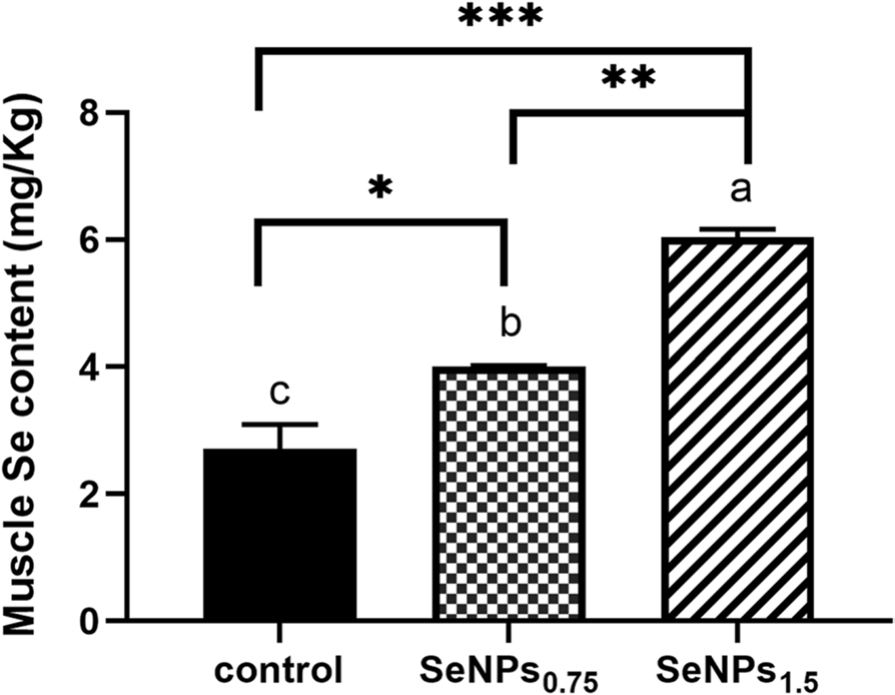
Se content in muscle tissue of Nile tilapia fed on different levels of SeNPs. Data were represented as Mean ± SEM (*n* = 3). Values with a different letter superscript are significantly different between groups (ANOVA with post hoc Tukey test, *P (< 0.05), **P (< 0.01). ***P (< 0.001)

Selenium content in fish diets was determined to be 0.22 mg/kg (a commercial diet used as a inorganic Se), 0.79 mg/kg (SeNPs_0.75_), and 1.8 mg/kg (SeNPs_1.5_). These values were marginally higher than the specified concentrations, owing to the presence of trace amounts of Se in these ingredients. Se content in fish musculature was increased by SeNPs supplementation in the fish diets (Fig. 2). It exhibited a dose-related increment, being significantly higher in fish fed 0.75 and 1.5 mg (*P* < 0.05) compared with the values of fish fed the control diet.

### Serum biochemical indices

**Fig. 3:**
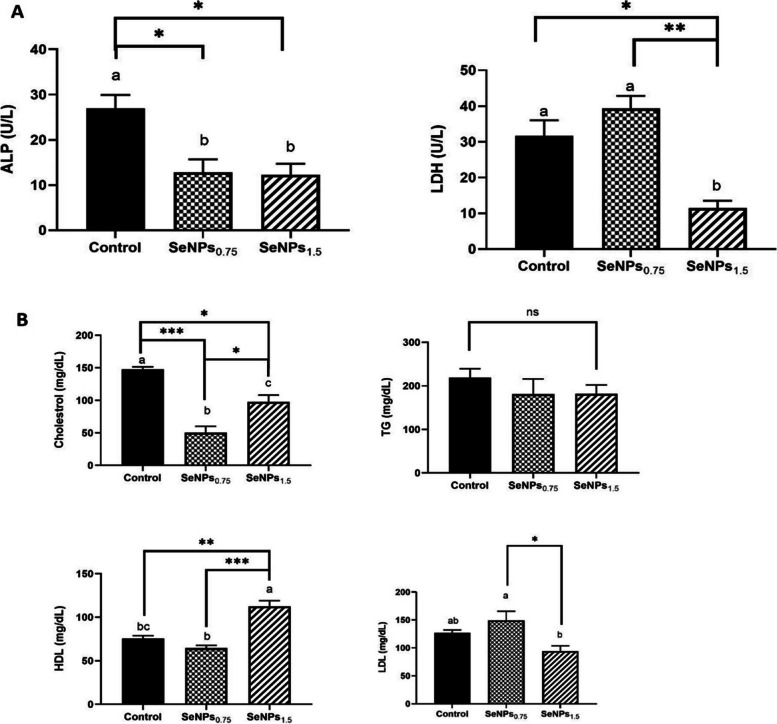
Serum biochemical indices of Nile tilapia supplemented with biosynthesized selenium nanoparticles (0, 0.75, and 1.5 mg SeNPs/kg) for 8 weeks (*N* = 6). **A** Liver enzymes activity, alkaline phosphatase (ALP) and lactate dehydrogenase (LDH). **B** Lipid profile, cholesterol, TG, LDL, and HDL. Data were expressed as Mean ± SEM. Values with a different letter superscript are significantly different between groups. Asterisks indicate levels of significance (ANOVA with post hoc Tukey test, *P < 0.05; **P < 0.01; ***P < 0.001)

ALP enzyme activity was significantly lower (over 1.5-fold decrease) in fish fed both doses of SeNPs (0.75 and 1.5 mg/kg Body weight) than in fish fed the control diet (*P* < 0.05). No significant alterations were observed in the activity of ALP between the two levels of SeNPs. Additionally, LDH enzyme had a notably lower value in the group of fish fed 1.5 mg SeNPs/kg compared to fish fed the basal diet (nearly threefold decrease, *P* < 0.05) and SeNPs_0.75_ fish group (over threefold decrease, *P* < 0.01), without statistical changes between the latter (*P* > 0.05) (Fig. 3A). Cholesterol levels were significantly lower in the SeNPs_0.75 & 1.5_ fish groups (nearly threefold decrease, *P* < 0.05, and 1.5-fold decrease, respectively). A marked decrease in cholesterol levels was observed in the SeNPs_0.75_ fish group compared to the SeNPs_1.5_ fish group (0.5- fold decrease, *P* < 0.05). No significant difference was observed in the values of Triglyceride (TG) between the control and SeNPs-treated groups (Fig. 3B). A notable increase in HDL levels was observed in the SeNPs_1.5_ fish group compared to the control (1.5-fold increase, *P* < 0.01) and SeNPs_0.75_ fish groups (nearly twofold increase, *P* < 0.001). Nevertheless, there was a notable decrease in LDL levels in the SeNPs_1.5_ fish group compared to that in the SeNPs_0.75_ fish group (nearly twofold increase, *P* < 0.05). However, no notable changes in LDL levels were detected compared with the control diet (Fig. 3B).

### Intestinal genes expression

**Fig. 4:**
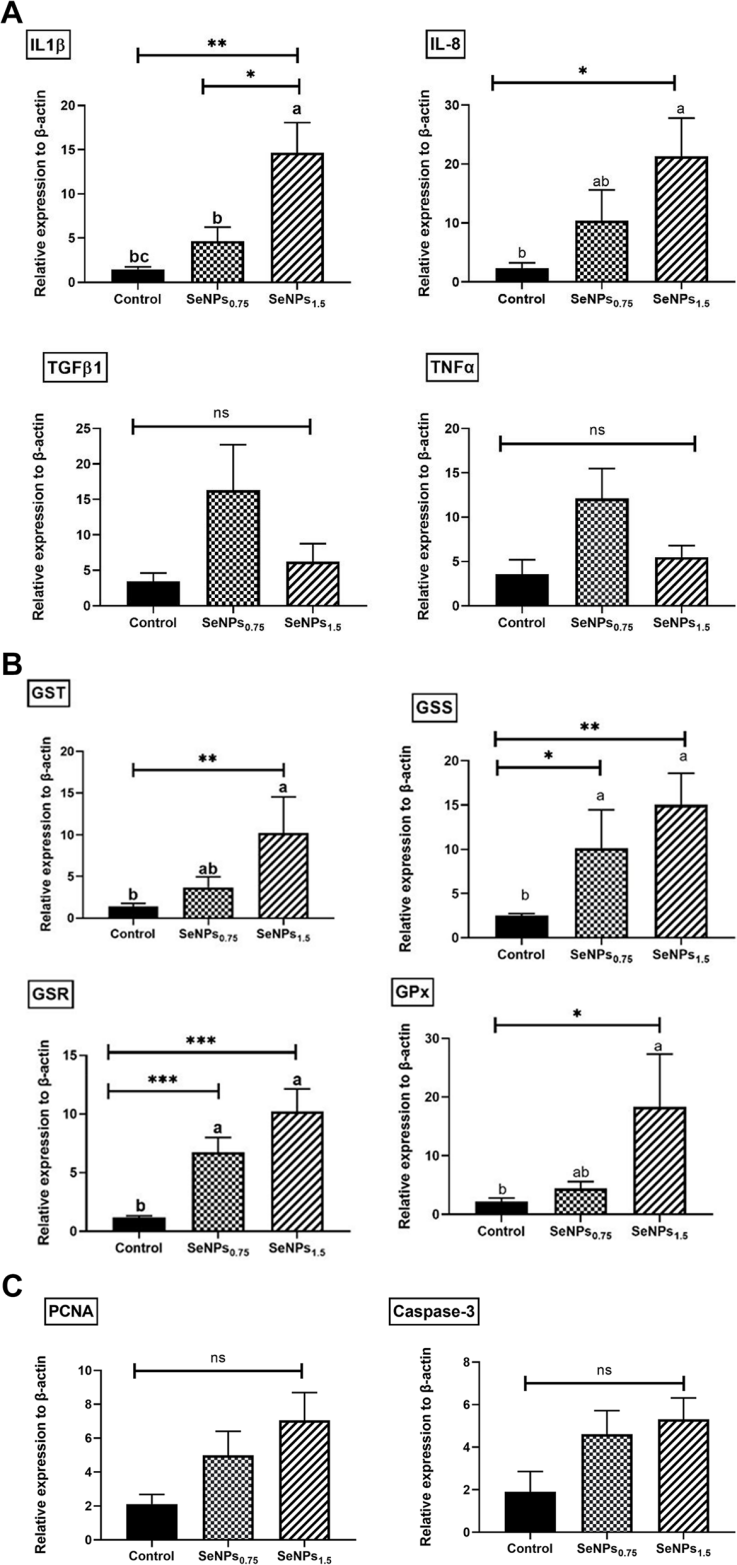
Comparative intestinal gene expression of (**A**) pro-inflammatory genes (e.g., *TNF-α*, *IL-8*, and *IL-1β*), and anti-inflammatory gene *(TGF-β*), (**B**) antioxidant genes (e.g., *GPx*, *GST*, *GSR*, and *GSS*), and (**C**) regulatory and apoptotic-related genes (PCNA and caspse-3) of Nile tilapia fed biosynthesized SeNPs (0, 0.75, and 1.5 mg SeNPs/kg) for eight weeks (*N* = 6). The qPCR detected transcript levels were normalized to the expression of a reference gene, Nile tilapia *β-actin*, and presented as Mean ± SEM. The values with a different letter superscript are significantly different between groups. Asterisks indicate levels of significance (ANOVA with post hoc Tukey test, *P < 0.05; **P < 0.01; ***P < 0.001)

SeNPs_1.5_ fish group exhibited a significant upregulation in the transcription of intestinal *IL-1β* and *IL-8* genes (ninefold increase, *P* < 0.01; tenfold increase, *P* < 0.05), respectively, compared to the control fish group (Fig. 4). Furthermore, *IL-1β* was significantly upregulated SeNPs_1.5_ fish group compared to that in the SeNPs_0.75_ fish group (threefold increase, *P* < 0.05) (Fig. 4A). However, *TNF-α* and *TGF-β* gene transcription showed no notable changes (*P* > 0.05) between the SeNPs and control fish groups (Fig. 4A). Concerning the antioxidant genes, the SeNPs_1.5_ fish group displayed significant upregulation of the intestinal *GST* (sevenfold increase,* P* < 0.01) and *GPx* (eightfold increase, *P* < 0.05) genes compared to fish fed the control diet. However, no significant changes were observed in other groups (Fig. 4B). Interestingly, the expression of intestinal *GSS* and *GSR* were in similar trend, where significant upregulations were noticed in SeNPs_0.75 & 1.5_ fish groups (fourfold increase, *P* < 0.05; sixfold increase, *P* < 0.01) in case of *GSS* and (sevenfold increase, *P* < 0.05; tenfold increase, *P* < 0.01) compared to the control (Fig. 4B). *PCNA* and *caspase*-3 gene expression exhibited no significant changes (*P* > 0.05) among groups (Fig. 4C).

### Histomorphometric analysis

**Fig. 5:**
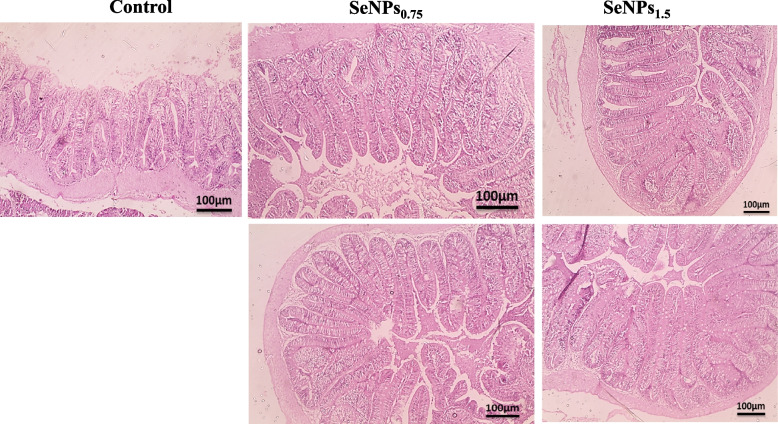
Photomicrographs of H&E-counter stained transverse sections from the intestine of non-supplemented Nile tilapia or supplemented with biosynthesized selenium nanoparticle (SeNPs) at SeNPs_0.75_ mg/Kg or SeNPs_1.5_ mg/Kg showing no structural damage. Low magnification (X10, bar 100 µm). Control = group fed basal diet; SeNPs_0.75_ = group fed basal diet with the addition of 0.75 mg/kg SeNPs; and SeNPs_1.5_ = group fed basal diet with the addition of 1.5 mg/kg SeNPs

**Fig. 6:**
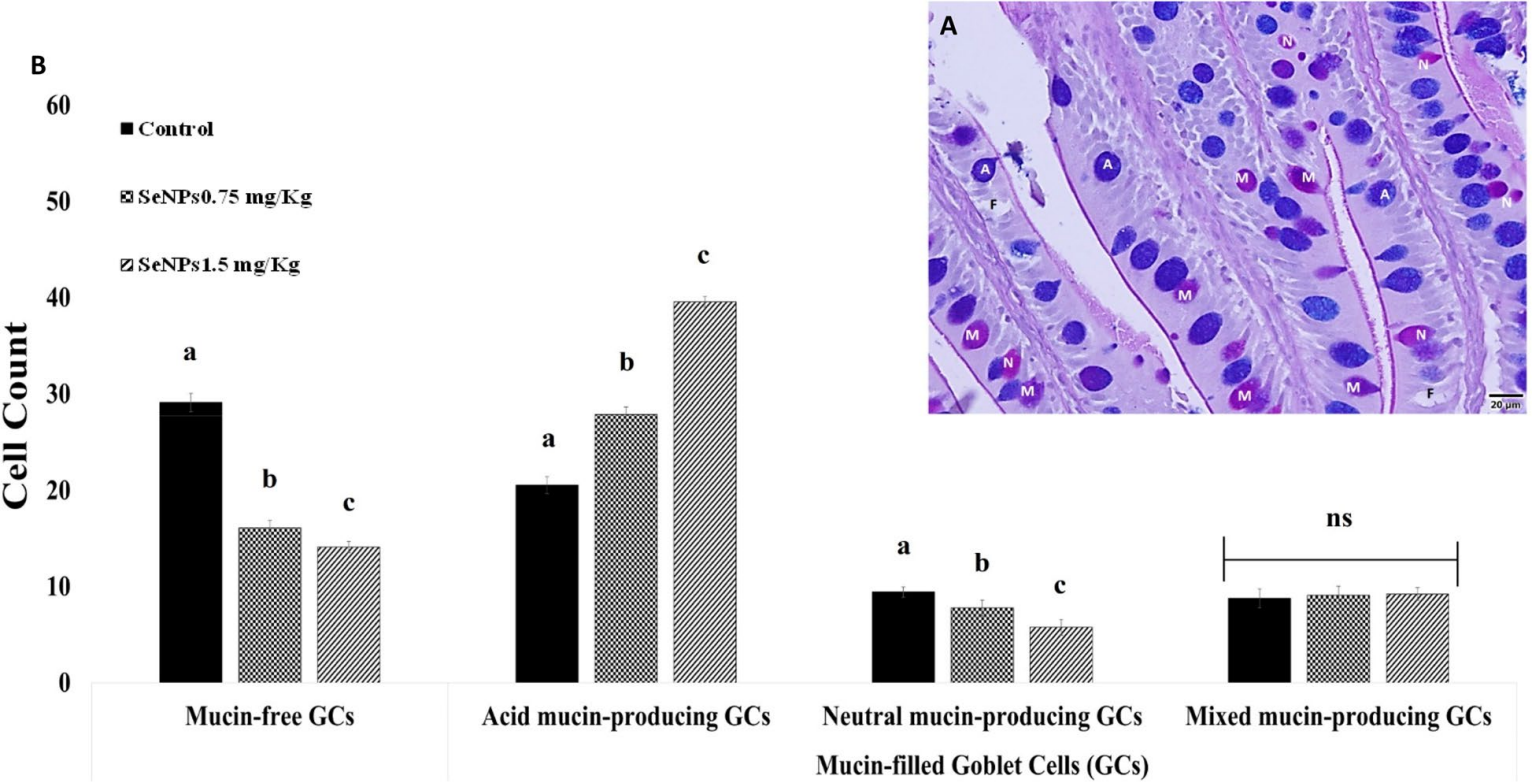
Differential count of the goblet cells (GCs) in the intestine of Nile tilapia fed on SeNPs_0.75_ mg/Kg, or SeNPs_1.5_ mg/Kg feed or basal diets for 8 weeks. **A**. AB & PAS double staining showing color differentiation of four types of the GCs, including mucin-free (negative stain), acid mucin-producing (blue, A), neutral mucin-producing (pink, N), and mixed mucin-producing cells (purple, M). **B** Bars demonstrate the statistical analysis of the intestinal goblet cells count of non-supplemented Nile tilapia or fed with SeNPs_0.75_ mg/Kg, or SeNPs_1.5_ mg/Kg supplemented diets. Data were expressed as Mean ± SEM. Values with a different letter superscript are significantly different between groups

No histopathologic lesions were detected in all groups of tilapia-fed basal diets or diets supplemented with SeNPs at either dose (Fig. 5). Likewise, the number of mucins-producing GCs significantly increased from 28.78 ± 0.83 in control fish group to 44.78 ± 0.97 and 54.56 ± 0.53 (*P* < 0.05) in the SeNPs_0.75 &1.5_ fish groups, respectively. In contrast, mucin-free GCs significantly decreased (*P* < 0.05) to 14.11 ± 0.6 in the intestines of the SeNPs_1.5_ fish group, and 16.11 ± 0.78 in the SeNPs_0.75_ fish group from 29.11 ± 0.93 of the control un-supplemented fish. Among GCs, acid mucin-producing GCs significantly increased (39.56 ± 0.53, *P* < 0.05) in the fish fed on SeNPs_1.5_ fish group, followed by the SeNPs_0.75_ fish group (27.89 ± 0.78), as compared with the control un-supplemented fish (20.56 ± 0.88), while the number of neutral mucin-producing GCs decreased significantly (5.78 ± 0.83, *P* < 0.05) in the intestines of the SeNPs_1.5_ fish group, followed by the SeNPs_1.5_ fish group (7.78 ± 0.83) compared to the control (9.44 ± 0.53). However, SeNPs supplementation did not significantly affect the number of mixed mucin-producing GCs (*P* > 0.19) (Fig. 6A & B).

## Discussion

Selenium nanoparticles have small particle size and large surface characteristics, which potentiate higher permeability and availability in the body of fish [6]. TEM analysis confirmed the green biosynthesis of SeNPs using *P. boryanum*, which could be used for the biological reduction and stabilization of selenium metal ions due to phenolic compounds found in this microalga: gallic, protocatechuic, chlorogenic, hydroxybenzoic, and vanillic [49], which have participated as biological reducing agents in salted ions and converted into nanoparticles, subsequently stabilizing these particles are marked by zeta potential values [5, 6, 50]. Thus, the green route for biosynthesis of nano-selenium from algal extracts is an economically viable mechanism that contributes to stable selenium nanoparticle formation [51]. The major site of fish digestion and immunity is the intestinal tract, which elucidates absorption and health status, as the intestine is widely related to the teleost intestinal immune barrier [52]. In the present study, little information was available on the effects of dietary microalgae derived SeNPs supplementation on the physiological, intestinal immune, and antioxidant capacities correlated with the histological parameters of Nile tilapia.

Se supplementation in a fish diet is essential to evaluate the optimum requirements for dietary Se levels to maintain the health status and subsequent a better stress resistance capability of fish [53]. In addition, commercial diets of cultured fish may not satisfy their demand for selenium because of the low availability of Se from fishmeal diets, as well as the effects of various environmental stressors in reared water [54]. In the present study, dietary inclusion of two concentrations of SeNPs (0.75 and 1.5 mg/kg) was provided to Nile tilapia to investigate the assimilation of Se in the muscle tissue and the overall effects on immune response in comparison with the normal inorganic Se source present in the mineral premix of the basal diets. In general, many studies have documented Se requirements of many fish species ranging from 0.2 to 12 mg/kg, which could be related to physiological changes in fish, Se concentrations in cultured water, time of exposure, and Se sources (organic, inorganic, or nano form) [53, 55, 56]. For Nile tilapia, dietary optimum levels of seleno-methionine were determined at 1.06–2.06 mg/kg diet for 10 weeks, having a beneficial effect on the tissue bioavailability and antioxidant enzymes activity, whereas higher dietary Se levels between 6.31–14.7 mg /kg diet revealed selenium toxicity via impairment in most of the physiological indices and retarded growth [57]. Furthermore, a previous study evaluated the optimum dietary Se requirement of tilapia at 1.23 mg nano-Se/kg feed for 90 days to enhance growth and expression of immune-related genes [58]. The Se concentrations of this study are in favor of those reports, and the optimum Se concentration was close to 1.0–2.0 mg/kg of Se, which was informed to provide a beneficial impact on tilapia and to avoid potential harmful outcomes from higher inclusion Se level in Nile tilapia.

Se levels in different fish tissues have been shown to be remarkable indices for evaluating the status and bioavailability of Se [57]. In particular, bioaccumulation of Se in fish fillets is important because of its prospective influence on consumers [59]. Likewise, it has been emphasized that Se supplementation in nanoparticle form in fish diets is more bioavailable and well-assimilated by fish than other sources of Se [58, 60]. According to our observations, Se concentrations in the musculature of Nile tilapia notably increased in a dose-dependent manner. Higher musculature Se concentrations with increasing dietary Se levels have been assayed in a variety of fish species [60–62]. Similarly, the musculature Se content of Nile tilapia fed 0.30 mg/kg Se for 10 weeks significantly increased [57]. Se content in the muscle tissue of Nile tilapia was significantly increased proportionally by dietary nano-Se supplementation (0.5, 0.1, 0.2 mg/kg) for 90 days [58]. These findings suggest that fish dietary nano-Se is efficient absorbed and bioavailability, that underscores the benefits of utilizing a Se-derived product to enhance Se levels.

Serum enzyme activities provide a critical evaluation of the health status of liver damage and cellular membranes of aquatic species [63]. Therefore, changes in serum biochemical parameters are frequently the first measurable indicators of ecological stress [64]. Se plays a vital role in regulating hepatic functions in the detoxification process, and biochemical indices are markedly influenced by a nutritionally balanced aquafeed and its content [65]. The present study showed a significant decrease in the serum levels of ALP in SeNPs_0.75_ and SeNPs_1.5_, while LDH was significantly reduced in SeNPs_1.5_, compared to the control group. Numerous studies have reported the effects of SeNPs on serum enzyme activities in various species. The first report indicated a marked reduction in serum AST, ALT, and ALP levels in Nile tilapias supplemented with a 0.7 mg/kg SeNPs diet for 9 weeks [8]. In addition, there was no significant difference in ALP serum activity among experimental common carp fed 0.5, 1, or 2 mg nano-Se/kg diets for 8 weeks [61]. However, this enzyme was markedly reduced in Nile tilapia fed 0.5, 1, and 2 mg nano-Se/kg diets for 90 d [66]. Our results are also similar to a previous report on common carp fed nano-selenium (0.7 mg/kg for 8 weeks, showing the lowest values of LDH compared with the control [67]. These results suggest that the fish were not stressed with the supplemented SeNPs doses and imposed no devastating effects on their hepatic health status.

As observed, cholesterol levels were significantly decreased in both SeNPs doses, while higher HDL levels were observed only in SeNPs_1.5_ fish group. These findings confirmed the potential role in regulation of lipid metabolism, where Se as antioxidant agent diminish the ROS production, which is required for the adipocyte-differentiation markers such as peroxisome proliferator-activated receptor (PPARγ), and thus disrupting with lipid deposition without cytotoxic effects [68]. Our findings were consistent with previous studies used nano-Se supplementation, like in Common carp fed on 2 and 0.7 mg nano-Se/kg for 8 weeks [9, 61], grass carp fed on at 0.3 mg/kg and 1.2 mg/kg [69], and Asian seabass (*Lates calcarifer*) fed Se on 4 mg/kg for four weeks [70]. On contrary, no significant differences were observed in total cholesterol and TG serum levels in Nile tilapia fed dietary chemically synthesized SeNPs (1 mg/kg) for two months [71]. These discrepancies could be related to different fish age, SeNPs dosage and synthesis method.

In the current study, upregulation of *IL-1β* and *IL-8* were observed, suggesting a better immune response after high-dose SeNPs supplementation, with no evidence of inflammatory changes as reflected by the mRNA levels of *TNF-α* and *TGF-β1*, coupled with normal histological intestinal morphometry in the present study. This finding implies that SeNPs potentially exert an immunomodulatory effect, that is indirectly related to its antioxidant activity reflected by upregulation of antioxidant-related genes expression. The glutathione family is required for strengthening the immune functions, including the proliferation of cells and activation of T cells and polymorphonuclear leukocytes in vivo [72]. In addition, As Se decreases ROS production, it inhibits the NF_k_β cascade [72, 73], with subsequent suppression of pro-inflammatory cytokines like *TNF-α* and *TGF-β1* as shown herein. In accordance with these findings, increased *IL-1β* and *IL-8* expression was observed after dietary supplementation of selenium-loaded chitosan nanoparticles (SeChNPs) in the liver and spleen of Nile Tilapia (*Oreochromis niloticus*) in a dose-dependent manner (0.5, 1, and 2 g/kg) [74]. Consistent with our findings, H Jingyuan, L Yan, P Wenjing, J Wenqiang, L Bo, M Linghong, Z Qunlang, L Hualiang and G Xianping [75] observed no significant alterations in the mRNA levels of *TGF-β1,* and *TNF-α* after dietary supplementation of different levels of selenium (0.10, 0.42, 0.67, 1.06 and 1.46 mg Se/kg) in juvenile blunt snout bream.

The expression of the antioxidant *GST*, *GSS*, *GSR*, and *GPx* genes was upregulated in the intestine of Nile tilapia-supplemented biosynthesized Se nanoparticles. Many reports have also positively elucidated the effect of SeNPs in enhancing the capacity of antioxidative enzymes (SOD, CAT, and GPx) in grass carp (*Ctenopharyngodon idella*) [69], Asian seabass [54], Nile tilapia [65, 76], common carp [9, 61], and European seabass (*Dicentrarchus labrax*) [77]. More specifically, Se nanoparticles can reinforce the intestinal antioxidant capacity, as elemental Se plays a pivotal role in building selenoproteins, functional components of GSH, and GPx enzymes, which prohibit cellular membrane peroxidation by catalyzing the removal of reactive oxygen species (ROS) in the fish body [11, 63, 78]. GPx-containing selenol is oxidized by H_2_O_2_ or other oxidants, which generates selenenic acid (GPx-SeOH). Subsequently, GPx-SeOH is converted into selenol. Subsequently, selenenyl sulfide (GPx-SeSG) is produced by the reaction between GPx-SeOH and GSH, which reduces GPx-SeSG to selenol [63, 79]. Additionally, nanoparticle forms of Se have been shown to promote GPx gene expression through the formation of selenophosphate [80]. Therefore, these enzymes have been noted as indicators of the effects of selenium on antioxidant mechanisms in fish [67]. Besides, the *P. boryanum* extracts showed the highest radical scavenging activity among *Chloromonas cf. reticulata* and *Chloroidium saccharophilum* microalgae due to the presence of Catechin, epicatechin, gallic acid, and vanillic phenolic compounds in *P. boryanum* microalga as natural antioxidants, neutralizing the reactive species of oxygen and nitrogen, subsequently prohibiting the lipid oxidative damage [81, 82]. Microalgal *P. boryanum* derived polysaccharides contribute to the modulation of antioxidant function regulation and increasing immunity response [49, 72].

As noted in the current study, the intestinal transcriptional levels of *PCNA* and *caspase*-3 in Nile tilapia remained unchanged after eight weeks of supplementation with biosynthesized SeNPs. Our findings are consistent with the protective effect of supplemented manganese nanoparticles (Mn-NPs) evidenced by the downregulation of caspase gene expression in *Pangasianodon hypophthalmus* fish [83], and in *Aeromonas*-challenged Nile tilapia dietary Se-loaded chitosan nanoparticles (0.5 g/kg), compared with control group [74]. The obtained data suggest a key role of Se in sustaining intestinal epithelial proliferation without apoptotic modifications, as previously documented [84], highlighting the role of SeNPs as powerful antioxidant agents, eliminating reactive oxygen species (ROS), which is linked to mitochondria-mediated apoptosis, *caspase*-3 activation, and cleavage of poly (ADP-ribose) polymerase-1 (*PARP*) [85].

Our investigations revealed a significant increase in the number of mucin-producing GCs in the intestine of Nile tilapia fed SeNPs-supplemented diets (SeNPs_1.5_ or SeNPs_0.75_) compared to that in control non-supplemented fish. The secreted intestinal mucin is made of glycoproteins and contains a number of bioactive molecules [86]. Intestinal mucin-filled GCs indicate mucin production, a potential component of the intestinal innate gut immune system [87]. In this study, a significant higher number of GCs producing acid mucins, which are sulfated intestinal mucins, was observed with no changes GCs producing mixed or neutral mucins. Sulfated mucins are resistant to lysis by host proteases and bacterial glycosidases thus conferring protection to the intestinal mucosa [47, 88], while neutral and mixed mucins participate in lubrication and osmoregulation [89]. Our results were consistent with those reported by S Ghaniem, E Nassef, AI Zaineldin, A Bakr and S Hegazi [90], who reported an increasing number of GCs in the anterior and posterior intestines of Nile tilapia fed SeNPs-supplemented diets (1 mg/kg diet) for 65 days. Upon integrating these findings with the intestinal cytokine and antioxidant-related gene expression, it is possible to deduce that SeNPs, as effective antioxidant agents, possess the capacity to mitigate intestinal inflammation and reduce the production of intestinal ROS. These effects are indirectly associated with the development of goblet cells and the promotion of mucus layer formation, which protects intestinal tissues [88, 91].

## Conclusions

In the current study, biochemical indices, Se bioavailability, expression patterns of intestinal antioxidant-related genes, *IL-8* and *IL1β* immune regulating genes, and goblet cell proliferation were enhanced by the incorporation of SeNPs in Nile tilapia diet, particularly at dose of 1.5 mg/kg diet. Further, SeNPs supplementation did not induce any damage as indicated by levels of PCNA and apoptotic genes expression. Therefore, incorporation of biogenic SeNPs into aquafeeds could potentially improve Nile tilapia immunity and sustainability.

## Data Availability

All data supporting the findings of this study are available within the paper.

## Abbreviations

AOAC: Association of official analytical chemists
DW: Distilled water
*GPx*: Glutathione peroxidase
*GSR*: Glutathione reductase
*GSS*: Glutathione synthetase
*GST*: Glutathione-S-transferase
GCs: Goblet cells
HDL: High-density lipoproteins
*IL-1β*: Interleukin-1β
*IL-8*: Interleukin 8
K: Condition factor
LDH: Lactate dehydrogenase
LDL: Low-density lipoproteins
MS-222: Tricaine methanesulfonate
NPs: Nanoparticles
NF-κB: Nuclear transcription factor-κB
NRC: National Research Council
*P. boryanum*: *Pediastrum boryanum*
ROS: Reactive oxygen species
SeNPs: Selenium nanoparticles
ALP: Serum alkaline phosphatase
SGR: Specific growth rate
TG: Triglycerides
*TGF-β1*: Transforming growth factor-β1
*TNF-α*: Tumor necrosis factor-*α*
TEM: Transmission electron microscopy
XRD: X-ray diffraction
